# Bioassay-Guided Isolation of an Anti-Ulcer Compound, Tagitinin C, from *Tithonia diversifolia*: Role of Nitric Oxide, Prostaglandins and Sulfhydryls 

**DOI:** 10.3390/molecules16010665

**Published:** 2011-01-17

**Authors:** María Elena Sánchez-Mendoza, Adelfo Reyes-Ramírez, Leticia Cruz Antonio, Luis Martínez Jiménez, Juan Rodríguez-Silverio, Jesús Arrieta

**Affiliations:** 1 Escuela Superior de Medicina, Instituto Politécnico Nacional, Plan de San Luis y Díaz Mirón, Colonia Santo Tomás, Delegación Miguel Hidalgo 11340, México D. F., Mexico; E-Mails: mesmendoza@hotmail.com (M.E.S.-M.); mtzj_luis@hotmail.es (L.M.J.); jrsilverio61@yahoo.com.mx (J.R.-S.); 2 Facultad de Estudios Superiores Zaragoza, Batalla del 5 de Mayo Esquina Fuerte de Loreto, Ejército de Oriente, México D.F., Mexico; E-Mails: adelfo@puma2.zaragoza.unam.mx (A.R.-R.); letycruza@yahoo.com.mx (L.C.A.)

**Keywords:** *Tithonia diversifolia*, Asteraceae, tagitinin C, gastroprotection, medicinal plants

## Abstract

*Tithonia diversifolia* is a medicinal plant from the Municipality of Suchiapa, Chiapas, Mexico, that according to local folk medicine is considered useful in the treatment of gastric ulcers. The aim of the present study was to investigate the gastroprotective activity of *T. diversifolia* by using an ethanol-induced gastric ulcer experimental model in male Wistar rats. The results showed that *T. diversifolia* had gastroprotective activity, and that the dichloromethane extract had the highest protective activity (close to 90% when using doses between 10 to 100 mg/kg), and that further the compound tagitinin C isolated from this extract was the main active gastroprotective agent. Rats treated with tagitinin C suspended in Tween 80 at 1, 3, 10 and 30 mg/kg showed 37.7, 70.1, 100, and 100% gastroprotection, respectively. The effect elicited by tagitinin C (30 mg/kg) was not attenuated by pretreatment with either *N*^G^-nitro-L-arginine methyl ester (70 mg/kg, i.p.), a nitric oxide (NO) synthase inhibitor, *N*-ethylmaleimide (10 mg/kg, s.c.), a blocker of sulfhydryl groups, or indomethacin (10 mg/kg, s.c.), a blocker of prostaglandin synthesis, which suggests that the gastroprotective mechanism of action of this sesquiterpene lactone does not involve NO, sulfhydryl groups or prostaglandins.

## 1. Introduction

Peptic ulcers represent one of the most important diseases of the digestive system and a medico-social problem of global economic importance, the latter due to its broad geographical distribution, as well as high incidence, morbidity and drug consumption. It is estimated that at some time in their life nearly 20% of all people may suffer from peptic ulcers, caused by factors such as stress, diet, smoking, alcohol and certain types of drugs [1].

The drugs currently used in the treatment of gastric ulcers are anti-acids, anticholinergics, proton pump inhibitors and H_2_-receptor antagonists. However, there are innumerable adverse effects caused by these allopathic medicines [2], indicating the need for more effective and safer antigastric ulcer agents with less side effects. In this context, metabolites derived from plants used in traditional medicine have provided an important basis for the discovery and development of modern therapeutic drugs [3].

*Tithonia diversifolia* (Hemsl.) A. Gray (Asteraceae) is a 2–5 m tall perennial shrub, which is native to Mexico and also grows in other countries of North America, and in parts of Africa and Asia [4]. *T. diversifolia* is administrated in several forms: oral decoction of the leaves for treatment of hepatitis, diabetes, malaria, pain, chemoprevention and anti-*Helicobacter pylori* [4,5,6], external application of dried leaves on wounds and infusion of leaves for the treatment of measles [6]. Previous phytochemical studies of this genus have shown that the major constituents include three subtypes of sesquiterpene lactones (heliangolides, furanoheliangolides and eudesmanolides), flavones, and chromenes, among others [7,8].

Although this plant is commonly used by the people of Suchiapa (Chiapas State, Mexico) to cure gastric ulcers, there is no scientific report either disproving or validating this therapeutic practice. Therefore, we decided to test the gastroprotective activity of *T. diversifolia* and, upon validating such protective action, proceeded to identify the active compound or compounds. A bioassay-guided fractionation was performed and the compounds obtained were tested in the absolute ethanol induced gastric ulcer experimental model in Wistar rats. The role of endogenous NO, sulfhydryl groups and prostaglandins was evaluated in relation to the gastroprotective effect in order to provide information about the mechanism of action of the test compounds. The results were compared with the effect of carbenoxolone.

## 2. Results and Discussion

### 2.1. Bioassay-guided fractionation and isolation of tagitinin C

Drug treatment of peptic ulcers has focused on either counteracting aggressive factors or stimulating the mucosal defenses. Although many drugs have been effectively employed in the treatment of gastroduodenal ulcers, all of these compounds have shown shortcomings, such adverse effects or high cost [9]. The search for new therapeutic options includes investigation of natural product sources [10]. In this context extracts of hexane, dichloromethane and methanol from the leaves of *Tithonia diversifolia* were evaluated. It was found that the dichloromethane extract was the most active (Table 1). Interestingly, this effect was not dose dependent. This extract presented a maximum gastroprotective effect (90.3 ± 1.9%) at 100 mg/kg, and similar values were obtained with doses of 10 and 30 mg/kg (80.6 ± 5.5% and 89.6 ± 2.5%, respectively).

**Table 1:** Gastroprotective effect of *Tithonia diversifolia* extracts on ethanol-induced ulceration in rats.

Treatment	Dose (mg/kg)	n	UI (mm^2^)	Gastroprotection (%)
Control	---	10	60.9 ± 14.1	---
Hexane extract	30	10	83.5 ± 10.9	-37.1 ± 7.8
	100	10	11.1 ± 3.6*	81.8 ± 5.9
Dichloromethane	10	10	11.8 ± 4.6*	80.6 ± 5.5
extract	30	10	6.3 ± 2.5*	89.6 ± 2.5
	100	10	5.9 ± 1.9*	90.3 ± 1.9
Methanol extract	30	10	44.1 ± 9.5	27.6 ± 9.5
	100	10	7.7 ± 1.7*	87.3 ± 3.2

*p < 0.05 *vs.* control group; UI = Ulcer index

**Figure 1:**
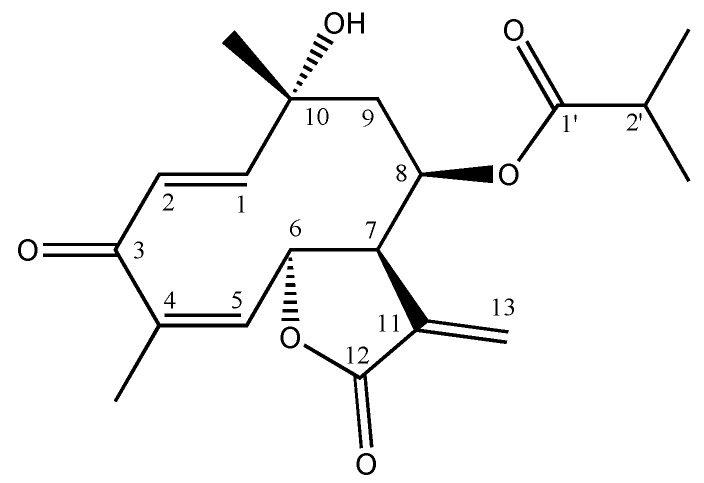
The structure of tagitinin C.

Thus, 70 g of the dichloromethane extract was subjected to separation over a silica gel column, and F2 was found to be the most active (maximum 98.7 ± 0.5% gastroprotective effect; see Table 2) among the five fractions obtained. Then 6.4 g of F2 were separated on a silica gel column and a pure compound from the F8’ fraction identified as tagitinin C (a sesquiterpene lactone), was obtained (Figure 1). The identity of this compound was confirmed by comparing its spectral data with that of the literature [11], and the gastroprotective activity of *T. diversifolia* was thus attributed to tagitinin C.

**Table 2:** Gastroprotective effect of the fractions of dichloromethane extract on ethanol-induced ulceration in rats.

Treatment	Dose (mg/kg)	n	UI (mm^2^)	Gastroprotection (%)
Control	---	8	76.6 ± 10.3	---
F1	30	8	32.0 ± 10.2*	58.2 ± 13.3
F2	30	8	0.9 ± 0.5*	98.7 ± 0.5
F3	30	8	19.5 ± 7.6*	74.5 ± 5.7
F4	30	8	23.5 ± 10.1*	69.4 ± 9. 2
F5	30	8	64.5 ± 7.7	15.8 ± 7.3

*p < 0.05 *vs.* control group; UI = Ulcer index

Tagitinin C was previously isolated from *T. diversifolia* [12] and many potential medicinal uses have been reported for this compound including as a cancer chemopreventive [13] and anti-malarial agent [12]. It has been reported that an aqueous extract of *T. diversifolia* obtained of leaves may be toxic when it is administrated consecutively in oral form (100 mg/kg) [6].

**Figure 2:**
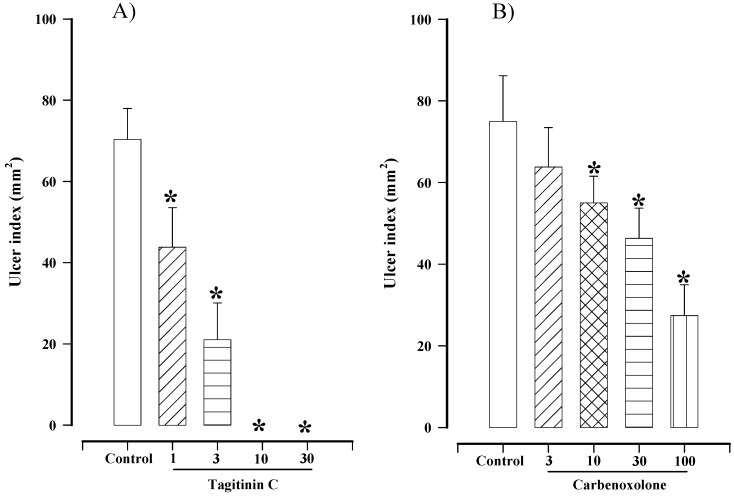
Effect of different doses of (A) tagitinin C (1–30 mg/kg) and (B) carbenoxolone (3–100 mg/kg) on gastric lesions induced in rats by absolute ethanol. Bars represent the mean ± SEM, n = 10. * p < 0.05 *vs.* the respective control; Dunn’s multiple comparison test after Kruskal-Wallis test.

The current study, representing the first report of its gastroprotective activity, found that tagitinin C produced a 100% gastroprotective effect at doses of 10 and 30 mg/kg (Figure 2A), and a maximum gastroprotective effect at the former dose. There are as yet no reports of toxicity for tagitinin C. For comparative reasons the effect of carbenoxolone was also studied, the maximal gastropotective effect induced by this compound being 63.5 ± 4.7% at a dose of 100 mg/kg (Figure 2B). 

### 2.2. Effect of L-NAME, indomethacin, and NEM on the gastroprotective effect

It is well known that NO is involved in the modulation of gastric mucosal integrity, and in the regulation of acid and alkaline secretion, mucus secretion and gastric mucosal blood flow [14]. In an attempt to provide information about the mechanism of the gastroprotective action of tagitinin C, the participation of NO was evaluated by pretreatment with L-NAME. 

**Figure 3:**
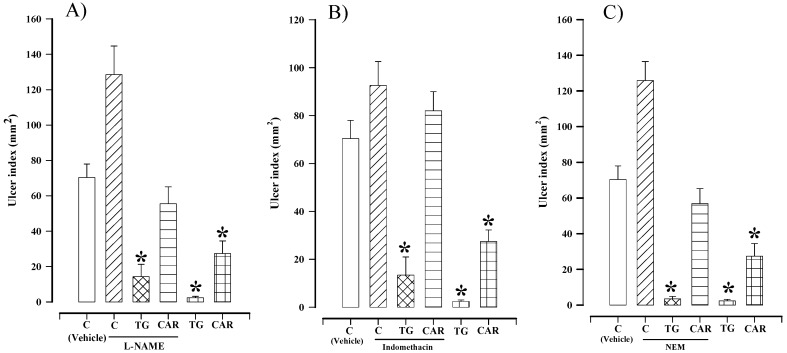
Effect of tagitinin C (TG) and carbenoxolone (CAR) at 30 mg/kg on gastric lesions induced by ethanol in rats pretreated with (A) L-NAME (70 mg/kg), (B) indomethacin (10 mg/kg) or (C) NEM (10 mg/kg). Bars represent the mean ± SEM, n = 10. * p < 0.05 *vs.* the respective control; Dunn’s multiple comparison test after Kruskal-Wallis test.

The ulcer index of the group treated with 70 mg/kg of L-NAME plus 30 mg/kg of tagitinin C was 14.3 ± 6.9 mm^2^ (Figure 3A), and this value was significantly different (p < 0.05) from the respective control treated with saline solution (70.3 ± 7.6 mm^2^). The current results show that in the presence of L-NAME the gastroprotective effect of tagitinin C was not inhibited, indicating that NO is not implicated in the action mechanism. Contrarily, NO does play a role in the mechanism of action of carbenoxolone (Figure 3A).

It is also well known that prostaglandins synthesized in large quantities by the gastrointestinal mucosa can prevent experimentally induced ulcers by ulcerogens. Thus, when the ulcer lesions are induced by absolute ethanol, the cytoprotective effect of the anti-ulcer agent can be mediated through endogenous prostaglandins [15].

Pretreatment with indomethacin (10 mg/kg) did not inhibit the gastroprotective action elicited by tagitinin C (13.4 ± 7.6 mm^2^), indicating that prostaglandins do not participate in the mechanism of action (Figure 3B). This value of gastroprotection was significantly different (p < 0.05) than the respective control using the vehicle (70.3 ± 7.6 mm^2^). Contrarily, the gastroprotective effect of carbenoxolone was attenuated (82.0 ± 7.9 mm^2^) by pretreatment with indomethacin (Figure 3B), indicating a role played by prostaglandins.

It has been demonstrated that the development of ethanol induced gastric damage is accompanied by a decrease in mucosal sulfhydryl compounds, due to the fact that these compounds are neutralized when they bind to the free radicals produced with tissue injury by noxious agents [16]. Aiming to investigate the possible role of endogenous sulfhydryl compounds involved in the gastroprotective effect promoted by tagitinin C, the animals were pretreated with NEM, a blocker of sulfhydryl compounds. This pretreatment (Figure 3C) did not inhibit the gastroprotective effect of tagitinin C (3.5 ± 1.3 mm^2^), as this value was significantly different (p < 0.05) with respect to the control group treated with the vehicle (70.3 ± 7.6 mm^2^). Therefore endogenous sulfhydryls are not involved in the gastroprotective effect. Contrarily, the gastroprotective effect of carbenoxolone was attenuated (56.9 ± 8.4 mm^2^) by pretreatment with NEM, indicating a role by sulfhydryls. 

The fact that a possible role of NO, sulfhydryl compounds, and prostaglandins was discarded in relation to the mechanism of gastroprotective action of tagitinin C, other possible mechanisms of action need to be investigated in future studies. Regarding carbenoxolone, the results of the present study are in agreement with data reported in the literature [17].

## 3. Experimental

### 3.1. General procedures

The ^1^H- and ^13^C-NMR spectra were recorded in a CDCl_3_ solution on a Bruker AVANCE (F) 300 spectrometer, working at 300 and 75 MHz, respectively. 

### 3.2. Plant material

The leaves of *Tithonia diversifolia* were collected in the Municipality of Suchiapa, Chiapas, during August of 2009. The plant was identified and registered by Francisco Hernández Najarro from the Flora Department of the Chip Herbarium, which is part of the Botanical Garden of the Secretary of Environmental Protection, Housing and Natural History of the State of Chiapas, Mexico. A specimen of the original collection can be found with the voucher number 42974.

### 3.3. Extraction and preliminary fraction

The leaves of *Tithonia diversifolia* were dried at room temperature (22 ± 2 °C) in the shade. After grinding 2.7 kg of leaves, compounds were successively extracted by maceration at room temperature (22 ± 2 °C) for 3 days, first with hexane (15 L × 3), then dichloromethane (15 L × 3) and finally methanol (15 L × 3). Evaporation of the solvents in vacuum yielded 80, 100 and 200 g of syrupy residues, respectively. The dichloromethane extract obtained from the leaves of *T. diversifolia* showed the most active gastroprotective effect (Table 1). Thus 70 g of this extract was subjected to percolation over a silica gel column (0.063–0.200 mm, 500 g) by using a step gradient of hexane/EtOAc (9:1, 1.5 L, F1), hexane/EtOAc (7:3, 1.5 L, F2), hexane/EtOAc (1:1, 1.5 L, F3), EtOAc (1.5 L, F4), and MeOH (1.5 L, F5). 29 g of fraction 2 (F2), which was the most active, were chromatographed on a silica gel column (290 g) by using a step gradient of hexane, hexane/EtOAc and EtOAc. From this procedure, fraction 3 (F3’) was the most active. Thus 6.4 g of this fraction were chromatographed on a silica gel column (64 g). Elution was performed with hexane and hexane/EtOAc mixtures, obtaining 24 fractions of 40 mL each. The main active gastroprotective agent from fraction 8 (hexane/EtOAc, 9:1) was identified as tagitinin C (500 mg) by comparison of the (^1^H and ^13^C NMR) spectral data with that of the literature [11].

### 3.4. Phytochemical data

Tagitinin C. ^1^H-NMR (CDCl_3_): d = 1.05 (3H, d, *J* = 6.9 Hz, CH_3_-C2’), 1.07 (3H, d, *J* = 6.9 Hz, CH_3_-C2’), 1.54 (3H, s, CH_3_-C10), 1.95 (3H, s br, CH_3_-C4), 2.02 (1H, dd, *J* = 14.1 and 4.2 Hz, H-9b), 2.42 (1H, dd, *J* = 14.1 and 4.2 Hz, H-9a), 2.42–2.52 (1H, m, H-2`), 2.6 (1H, br, OH), 3.54–3.58 (1H, m, H-7), 5.30–5.38 (1H, m, H-8), 5.41 (1H, d br, *J* = 9.0 Hz, H-6), 5.81 (1H, d, *J* = 1.8 Hz, H-13b), 5.87 (1H, d br, *J* = 9.0 Hz, H-5), 6.25 (1H, d, *J* = 17.1 Hz, H-2), 6.35 (1H, d, *J* = 1.8 Hz, H-13a), 6.94 (1H, d, 17.1 Hz, H-1). ^13^C-NMR (CDCl_3_): δ = 18.58 (CH_3_-C2’), 18.76 (CH_3_-C2’), 19.63 (CH_3_-C4), 28.95 (CH_3_-C10), 30.01 (C-2`), 47.00 (C-7), 48.36 (C-9), 71.99 (C-10), 73.95 (C-8), 75.97 (C-6), 124.49 (C-13), 129.60 (C-2), 136.02 (C-11), 137.12 (C-5), 138.89 (C-4), 160.16 (C-1), 169.69 (C-12), 176.21 (C-1’), 196.77 (C-3). These data match those in the literature [11]. 

### 3.5. Animals

All the experiments were performed with male Wistar rats, weighing 180–220 g, obtained from the animal house of the Superior Medicine School (IPN). Procedures involving animals and their care were conducted in conformity with the Mexican Official Norm for Animal Care and Handling (NOM-062-ZOO-1999), and in compliance with international rules on care and use of laboratory animals. Unless otherwise specified, the rats were placed in single cages with wire-net floors and deprived of food 24 h before experimentation, but allowed free access to tap water throughout. All experiments were carried out using 8–10 animals per group.

### 3.6. Drugs and dosage

Carbenoxolone (Sigma-Aldrich Co.) was used as the gastroprotective reference drug. The drugs were prepared freshly each time, suspended in 0.5% Tween 80 and administered by the intragastric route. Control rats received the vehicle (0.5% Tween 80) in the same volume (0.5 mL/100 g) and by the same route. *N*^G^-nitro-L-arginine methyl ester (L-NAME), *N*-ethylmaleimide (NEM) and indomethacin (IND) were purchased from Sigma Chemical Co. USA.

### 3.7. Acute gastric ulcer induced by absolute ethanol

A gastric ulcer was induced by administering absolute ethanol orally (1 mL) [17]. The extracts or drugs were administered to different groups 30 min before ethanol administration. Two hours after ethanol administration, the animals were sacrificed in a CO_2_ chamber. The stomach and duodenum were dissected out, inflated with 10 mL of formalin, and then placed in 2% formalin for 5 min to fix both the inner and outer layers. The duodenum was opened along its anti-mesenteric side and the stomach along the greater curvature. The damaged area (mm^2^) was measured under a dissection microscope (×10) with an ocular micrometer. The sum of the area of all the lesions in the corpus of each animal was calculated and served as the ulcer index. Gastroprotection (%) was calculated according to: % gastroprotection = (UIC-UIT) × 100/UIC, where UIC is the ulcer index in control and UIT is the test animal index [17].

### 3.8. Ethanol-induced gastric mucosal lesions in L-NAME pretreated rats

To investigate the involvement of endogenous NO in the gastroprotective effects of the compounds, L-NAME (70 mg/kg dissolved in saline solution) was intraperitonally injected to 3 experimental groups 30 min before the administration of either the vehicle, tagitinin C (30 mg/kg) or carbenoxolone (100 mg/kg) by the oral route [18]. Absolute ethanol was given to each rat in these groups 30 min later, and the animals were sacrificed 2 h after the administration of ethanol to measure the ulcer index. Two control groups (L-NAME-treated and non-L-NAME-treated) were included in this evaluation. 

### 3.9. Ethanol-induced gastric mucosal lesions in indomethacin pretreated rats

To investigate the involvement of endogenous prostaglandins in the gastroprotective effect of the compounds, a control group received a subcutaneous injection of 5 mM NaHCO_3_ in saline solution and another group an injection of indomethacin (10 mg/kg dissolved in NaHCO_3_ 5 mM) by the same route [18]. After 75 min, the animals in each of these two groups received one of three oral treatments (saline solution, 30 mg/kg tagitinin C or 100 mg/kg carbenoxolone). Absolute ethanol was given to each rat 30 min after tagitinin C or carbenoxolone administration and the rats were sacrificed 2 h later in a CO_2_ chamber. The stomachs were subsequently removed to measure the ulcer index, as aforementioned.

### 3.10. Ethanol-induced gastric mucosal lesions in NEM pretreated rats

To investigate the involvement of the endogenous sulfhydryls in the protective effects of tagitinin C and carbenoxolone, NEM (10 mg/kg dissolved in saline solution) was subcutaneously injected in 3 groups of animals 30 min before the oral administration of either the vehicle, tagitinin C (30 mg/kg) or carbenoxolone (100 mg/kg) [18]. Absolute ethanol was given to each rat 30 min later and rats were sacrificed 2 h after the administration of ethanol to measure the intensity of the gastric ulcer. Two control groups (NEM-treated and non-NEM-treated) were included in this experiment.

### 3.11. Statistics

Data are presented as the mean ± SEM from 8–10 rats per group. Statistically significant differences between the treatments were tested by the Kruskal-Wallis test followed by Dunn’s multiple comparison tests. Probability (p) values less than 0.05 were considered significant.

## 4. Conclusions

The current study demonstrates the effectiveness of *Tithonia diversifolia* in the treatment of gastric ulcer. Tagitinin C was identified as the main active gastroprotective agent in this traditional medicinal plant. The mechanism of the gastroprotective action shown by tagitinin C is not related to endogenous NO, prostaglandins or sulfhydryl groups. Further pharmacological investigations are necessary to provide evidence about the gastroprotective mechanism of action of tagitinin C.

## References

[1] Bucciarelli A., Minetti A., Milczakowskyg C., Skliar M. (2010). Evaluation of gastroprotective activity and acute toxicity of *Solidago chilensis* Meyen (Asteraceae). Pharm. Biol..

[2] Babu T.H., Manjulatha K., Kumar G.S., Hymavathi A., Tiwari A.K., Purohit M., Rao J.M., Babu K.S. (2010). Gastroprotective flavonoid constituents from *Oroxylum indicum* Vent. Bioorg. Med. Chem. Lett..

[3] Santin J.R., Lemos M., Klein L.C., Niero R., de Andrade S.F. (2010). Antiulcer effects of *Achyrocline satureoides* (Lam.) DC (Asteraceae) (Marcela), a folk medicine plant, in different experimental models. J. Ethnopharmacol..

[4] Kuroda M., Yokosuka A., Kobayashi R., Jitsuno M., Kando H., Nosaka K., Ishii H., Yamori T., Mimaki Y. (2007). Sesquiterpenoids and flavonoids from the aerial parts of *Tithonia diversifolia* and their cytotoxic activity. Chem. Pharm. Bull..

[5] Castillo-Juárez I., González V., Jaime-Aguilar H., Martínez G., Linares E., Bye R., Romero I. (2009). Anti-*Helicobacter pylori* activity of plants used in Mexican traditional medicine for gastrointestinal disorders. J. Ethnopharmacol..

[6] Adebayo J.O., Balogun E.A., Oyeleke S.A. (2009). Toxicity study of the aqueous extract of *Tithonia diversifolia* leaves using selected biochemical parameters in rats. Phcog Res..

[7] Yemele Bouberte M., Krohn K., Hussain H., Dongo E., Schulz B., Hu Q. (2006). Tithoniamarin and tithoniamide: a structurally unique isocoumarin dimer and a new ceramide from *Tithonia diversifolia*. Nat. Prod. Res..

[8] Ambrósio S.R., Oki Y., Heleno V.C., Chaves J.S., Nascimento P.G., Lichston J.E., Constantino M.G., Varanda E.M., da Costa F.B. (2008). Constituents of glandular trichomes of *Tithonia diversifolia*: Relationships to herbivory and antifeedant activity. Phytochemistry..

[9] Toma W., Gracioso J.S., de Andrade F.D., Hiruma-Lima C.A., Vilegas W., Souza-Brito A.R. (2002). Antiulcerogenic activity of four extracts obtained from the bark wood of *Quassia amara* L. (Simaroubaceae). Biol. Pharm. Bull..

[10] Al-Mofleh I.A., Alhaider A.A., Mossa J.S., Al-Sohaibani M.O., Al-Yahya M.A., Rafatullah S., Shaik S.A. (2008). Gastroprotective effect of an aqueous suspension of black cumin *Nigella sativa* on necrotizing agents-induced gastric injury in experimental animals. Saudi J. Gastroenterol..

[11] Baruah N.C., Sharma R.P., Madhusudanan K.P., Thyagarajan G., Herz W., Murari R. (1979). Sesquiterpene lactones of *Tithonia diversifolia*. Stereochemistry of the tagitinins and related compounds. J. Org. Chem..

[12] Goffin E., da Cunha A.P., Ziemons E., Tits M., Angenot L., Frederich M. (2003). Quantification of tagitinin C in *Tithonia diversifolia* by reversed-phase high-performance liquid chromatography. Phytochem. Anal..

[13] Gu J.Q., Gills J.J., Park E.J., Mata-Greenwood E., Hawthorne M.E., Axelrod F., Chavez P.I., Fong H.H.S., Mehta R.G., Pezzuto J.M., Kinghorn A.D. (2002). Sesquiterpenoids from *Tithonia diversifolia* with potential cancer chemopreventive activity. J. Nat. Prod..

[14] Chandranath S.I., Bastaki S.M.A., Singh J. (2002). A comparative study on the activity of lansoprazole, omeprazole and PD-136450 on acidified ethanol- and indomethacin- induced gastric lesions in the rat. Clin. Exp. Pharmacol. Physiol..

[15] Yamamoto K., Kakegawa H., Ueda H., Matsumoto H., Sudo T., Miki T., Satoh T. (1992). Gastric cytoprotective anti-ulcerogenic actions of hydroxychalcones in rats. Planta Med..

[16] Maity S., Vedasiromoni J.R., Ganguly D.K. (1998). Role of glutathione in the antiulcer effect of hot water extract of black tea (*Camellia sinensis*). Jpn. J. Pharmacol..

[17] Sánchez-Mendoza M.E., Reyes-Trejo B., Sánchez-Gómez P., Rodríguez-Silverio J., Castillo-Henkel C., Cervantes-Cuevas H., Arrieta J. (2010). Bioassay-guided isolation of an anti-ulcer chromene from *Eupatorium aschenbornianum*: Role of nitric oxide, prostaglandins and sulfhydryls. Fitoterapia..

[18] Reyes-Trejo B., Sánchez-Mendoza M.E., Becerra-García A.A., Cedillo-Portugal E., Castillo-Henkel C., Arrieta J. (2008). Bioassay-guided isolation of an anti-ulcer diterpenoid from *Croton reflexifolius*: role of nitric oxide, prostaglandins and sulfhydryls. J. Pharm. Pharmacol..

